# Mimickers of hypoxic-ischaemic brain injury in term neonates: What the radiologist should know

**DOI:** 10.4102/sajr.v28i1.2810

**Published:** 2024-02-29

**Authors:** Shalendra K. Misser, Moherndran Archary

**Affiliations:** 1 Faculty of Radiology, Lake Smith and Partners Inc., Durban, South Africa; 2 Department of Radiology, Faculty of Health Sciences, Nelson R Mandela School of Medicine, University of KwaZulu-Natal, Durban, South Africa; 3 Department of Pediatrics, Faculty of Health Sciences, Nelson R Mandela School of Medicine, University of KwaZulu-Natal, Durban, South Africa

**Keywords:** hypoxic-ischaemic, cerebral palsy, term neonatal, magnetic resonance imaging, neurometabolic

## Abstract

Patterns of neonatal hypoxic-ischaemic brain injury (HIBI) are fairly well known. There are, however, other diagnoses with imaging patterns that may mimic HIBI. A review of MRI studies was conducted for children with suspected cerebral palsy, correlated with prior imaging, clinical details and laboratory tests where available. In the 63 identified cases, imaging features were, in many cases, very similar to the known patterns of HIBI. The alternative diagnoses can be classified as developmental, vascular, chromosomal, infections, metabolic disorders, and congenital syndromes. These findings are described in this pictorial essay. The potential mimickers of HIBI described in this essay can demonstrate similar imaging appearances to HIBI.

**Contribution:**

There are multiple possible causes of neonatal encephalopathy other than hypoxic-ischaemic encephalopathy. Many conditions may mimic HIBI, each of which can be associated with significant morbidity. It is prudent for the reporting radiologist to be aware of these alternate clinico-radiological diagnoses.

## Purpose

**FIGURE 1:**
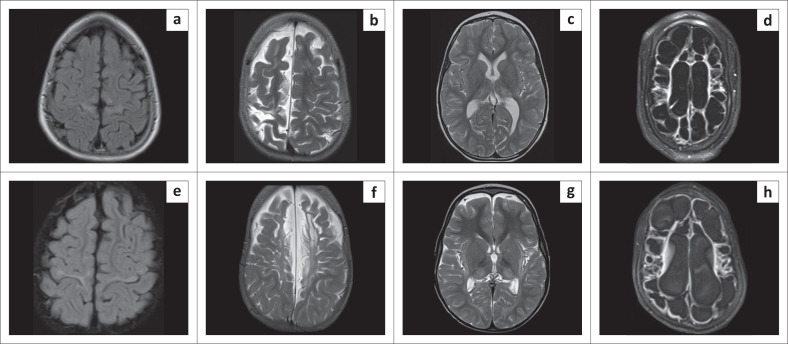
Blinded to clinical information, it is not always possible to distinguish hypoxic-ischaemic brain injury from its mimicker (Can you guess which are cases of hypoxic-ischaemic brain injury?).

Cerebral palsy (CP) is the most common motor disorder of childhood globally.^1^ In recent years, there has been a marked increase in medicolegal litigation pertaining to CP from perinatal hypoxic-ischaemic encephalopathy (HIE). Radiologists have become key figure in identifying patterns of brain injury on MRI that are indicative of hypoxic-ischaemic brain injury (HIBI). While these patterns^2^ are fairly well known, it is also important for the reporting radiologist to be aware of other diagnoses with imaging patterns that may mimic HIBI. To highlight the variability of patterns of HIBI and the overlap of these patterns with other causes, we have hidden (in Figure 1) the diagnoses of each child imaged. Even the most experienced paediatric neuroradiologists have difficulty in correctly assigning the diagnosis to these cases, let alone separating them into those cases with HIBI and those with alternative causes. The correct diagnoses is revealed in the conclusion of this pictorial essay in Figure 34.

## Background

There are typical features that the reporting radiologist seeks to identify while reviewing an MRI study performed for neonatal encephalopathy (NE) to suggest a diagnosis of perinatal term HIBI. The classic patterns of injury generally alluded to in daily reporting include the central, watershed or parasagittal, combined central and watershed (mixed pattern), and cystic encephalomalacia.^2^ While HIE may be one of the commonest causes of perinatal encephalopathy, and accounting for 46% of cases of neonatal seizures,^3^ it must be observed that there are several other scenarios resulting in a similar constellation of clinical findings that may mimic the clinical diagnosis of HIE. Furthermore, it has been reported that only 14.5% of CP cases were associated with an intrapartum event^4^; yet, most medicolegal litigation cases are based on the premise of intrapartum negligence. An MRI serves a vital role in clarifying the imaging features that may indicate a condition mimicking HIE. This is critical in the evaluation of children with suspected medical negligence, who are imaged several months or years after birth. The radiologist is often the lead expert, providing supportive proof in describing the diagnostic MRI patterns. These patterns are still well-recognised in the chronic phase of evolution, long after delivery.^5^ An MRI should be undertaken whenever possible in CP cases, even in the absence of prior perinatal imaging. It was shown that out of a database of 1620 CP children, MRI could possibly avoid litigation in up to 31% of cases.^5^

## Methods

A database of MRI studies of CP patients with clinically suspected term neonatal HIBI was accumulated. Some were imaged in the neonatal period. Other scans were acquired months to years later to assess for stigmata of HIBI. These MRI studies were all performed on 1.5T scanners at the imaging centres of Lake Smit and Partners Inc. Sequences obtained in all patients were previously enumerated.^2^ All MRI studies of these full-term infants were retrospectively reviewed by the principal investigator. Of the composite database, we identified numerous cases where, although the pattern of cerebral injury identified on the MRI studies could be attributed to HIBI, we recorded the clinical and follow up investigations to arrive at a final clinical diagnosis. The data pertaining to these children were extracted from available clinical notes and in some instances, by confirmation from the clinicians involved in the management of the patients.

## Results

**FIGURE 2:**
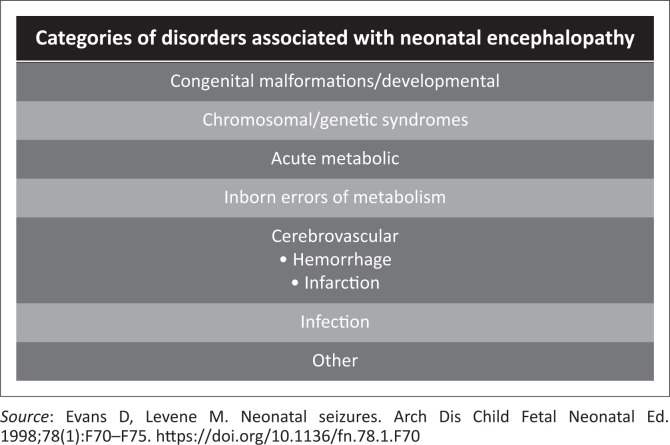
Categories of disorders associated with neonatal encephalopathy.

There were 25 male patients and 38 female patients in this study. The youngest child imaged was 2 days old and the oldest 15 years of age. There were numerous diagnoses reported, verified clinically, explaining the NE and accounting for the presentation other than due to HIE. Metabolic causes or inborn errors of metabolism were identified in 12 cases, congenital malformations in 10 cases, cerebrovascular causes in 8 cases, infection related changes in 20 cases, chromosomal and other causes accounting for the remaining cases. Following from the categorisation of final diagnoses, we established the differential diagnosis of NE, to include congenital malformations, inborn errors of metabolism, central nervous system (CNS) infections, severe trauma and cerebrovascular syndromes, as shown in Figure 2.^3^

### Congenital malformations

**FIGURE 3:**
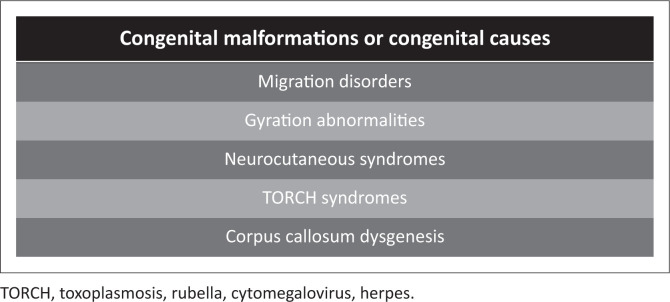
Congenital malformations or congenital causes that may mimic hypoxic-ischaemic encephalopathy usually because of perinatal seizures.

A myriad of congenital malformations are known with the potential to develop a clinical syndrome depicting CP and these are sometimes attributed, often erroneously, to perinatal HIE. These malformations develop in utero and many of them manifest with neonatal or infantile seizures. Although some neonates can appear encephalopathic, there is generally a lack of supportive features of HIE such as metabolic acidosis. Cord blood gas analyses have been recommended to confirm neonatal acidosis.^6^ Despite this lack of supportive clinical context, several cases are referred for medicolegal evaluation to exclude features of HIBI. The MRI studies are often quite instructive in excluding HIBI because of the clear depiction of specific malformations. Some of these are listed in Figure 3.

**FIGURE 4:**
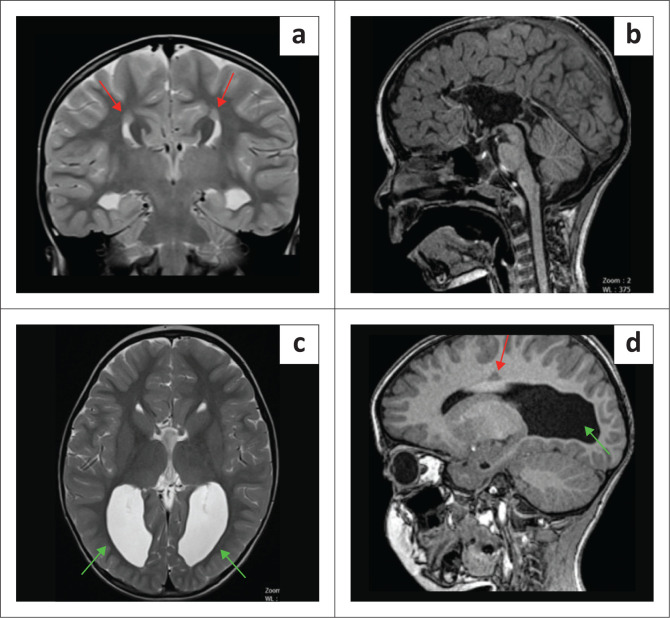
(a-d) A 5-year-old male child with a history of neonatal seizures for medicolegal evaluation of hypoxic-ischaemic brain injury. The absence of the corpus callosum with a steer-horn configuration of the frontal horns, colpocephaly (green arrows) and parallel lateral ventricle bodies. Note the grey matter heterotopia as annotated (red arrows) superior to both lateral ventricles.

**FIGURE 5:**
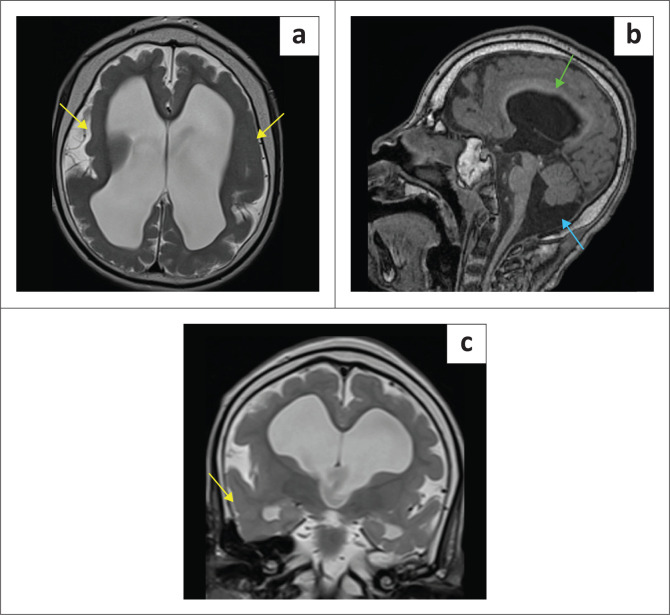
(a-c) Nine-year-old male with neonatal and ongoing seizures through childhood, being assessed for possible term neonatal hypoxic-ischaemic brain injury. The MRI shows pachygyria associated with a smooth cortical surface (yellow arrows), figure-of-eight configuration, dysplastic corpus callosum (green arrow), inferior vermian atrophy and posterior fossa retrocerebellar cyst (blue arrow) – features of Miller-Dieker Syndrome due to *LIS1* mutation.

Corpus callosum agenesis is one of the commonest malformations identified in clinical radiology and may be categorised as a partial or total dysgenesis.^7^ These may be associated with other brain malformations (hindbrain, heterotopia or cortical dysplasia) as in the patient presented in Figure 4. Malformations of cortical development, gyration abnormalities (Figure 5), neuronal migration abnormalities and posterior fossa cysts, are among several other malformations that may be clinically similar with encephalopathic neonates; yet, the key would be the lack of recorded perinatal metabolic acidosis in these instances. Pontocerebellar hypoplasia, for instance, may mimic dyskinetic or ataxic CP. Dedicated early genetic testing has been recommended^8^ in identifying potential mimickers of CP in around one-third of cases.

### Neurometabolic disorders

**FIGURE 6:**
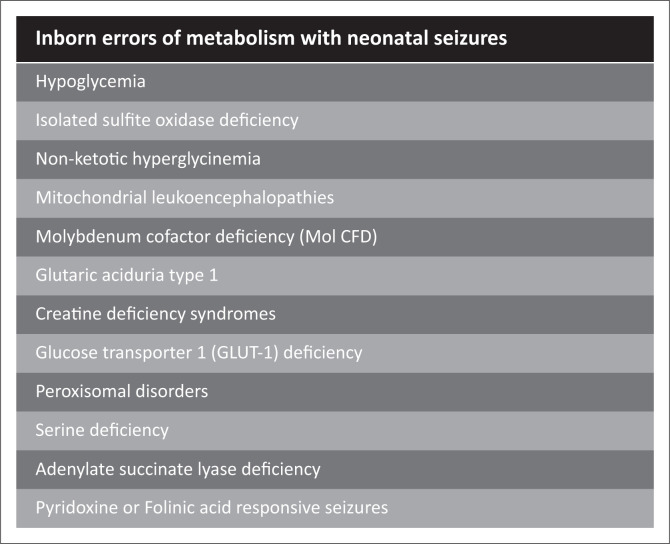
Inborn errors of metabolism that can present with neonatal seizures and may be differential considerations for neonatal encephalopathy.

The approach to neurometabolic disorders envisages two broad presentations: one with a relatively normal neonatal period, and the second with NE.^9^ Metabolic disorders that manifest in the neonatal period are often devastating.^10,11^ Many metabolic disorders may present with neonatal seizures, as enumerated in Figure 6, making these important differential considerations for NE.^12^ These are distinguishable from those inborn errors of metabolism that have a relatively normal neonatal period or the disorder may be masked by the support provided by the mother and placenta in the late gestational period before delivery.^11^

**FIGURE 7:**
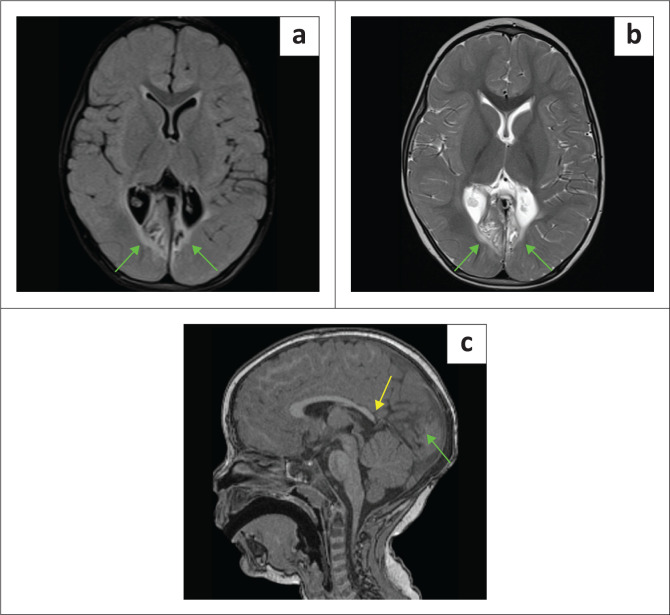
(a-c) *Neonatal hypoglycaemia* with bilateral medial occipital lobe encephalomalacia (green arrows) in a 20-month-old female child. Note the corpus callosum thinning with splenium (yellow arrow) atrophy.

An important metabolic cause of NE is neonatal hypoglycaemia (Figure 7), which has a very similar clinical presentation to HIE, and can be indistinguishable from the parasagittal watershed pattern of HIBI on imaging because of partial prolonged pathophysiology. There is commonly posterior parietal and occipital lobe spongiosis, but global brain injury may also be seen in association with seizures.

The thalamus L-sign^13^ described in 2022 is a useful MRI finding distinguishing pure perinatal hypoglycaemia from HIBI or combined HIBI/hypoglycaemia. In the series of 297 cases, the thalamus L-sign was shown as a potential biomarker for the parasagittal/partial prolonged subtype of HIBI, noticed in 86% of such patients. None of the patients with pure hypoglycaemia demonstrated this sign (Figure 7).

**FIGURE 8:**
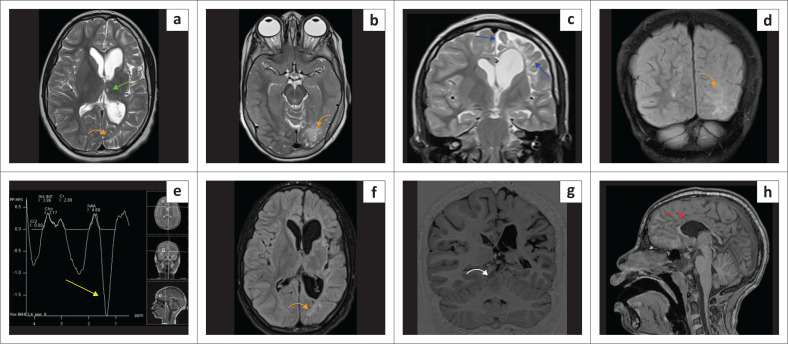
(a-h) A 9-year-old female with epilepsy and stroke-like episodes was sent for imaging to exclude features of hypoxic-ischaemic brain injury. Note left occipital posterior cerebral artery territory infarct (orange arrows), left thalamic paramedian lacune (green arrow), left frontoparietal parasagittal anterior cerebral artery territory infarction (blue arrows), and magnetic resonance spectroscopy (MRS) showing a deep lactate peak (yellow arrow) on short time to echo (TE) because of mitochondrial encephalomyopathy, lactic acidosis and stroke-like episodes (MELAS). There is also superior cerebellar encephalomalacia (white arrow) from a prior infarct and marked thinning of the corpus callosum (red arrow).

Of the metabolic aetiologies, lactic acidosis can be one of the commonest features (e.g. because of mitochondrial disorders – Figure 8 – Figure 10) identified on imaging using magnetic resonance spectroscopy (MRS). Urea cycle disorders, amino acidurias (including maple syrup urine disease [Figure 11]) and molybdenum co-factor deficiency/sulfite oxidase deficiency (Figure 12) are others to consider. Genomic assessment in CP children is crucial in identifying neurogenetic conditions masquerading as HIBI. This is achieved using chromosomal microarray and whole exome/genome sequencing, with identification of specific genetic mutations. Although each genetic and metabolic cause may be individually rare, collectively, these are clinically important masqueraders of CP.^14^ Leach et al.^15^ described, through a systematic literature review, 54 treatable inborn errors of metabolism that can mimic CP.

**FIGURE 9:**
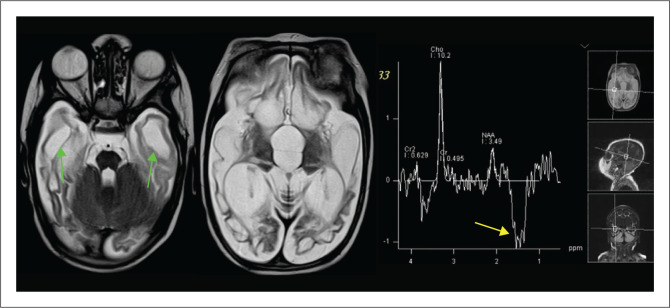
An MRI was performed on an 11-month-old child who had neonatal encephalopathy. There is cystic encephalomalacia at the temporal lobes (green arrows). The magnetic resonance spectroscopy (MRS) demonstrates marked suppression of the N-acetylaspartate peak, relative elevation of the choline peak and prominent lactate peak (yellow arrow). The combination of findings is indicative of a *mitochondrial disorder*.

**FIGURE 10:**
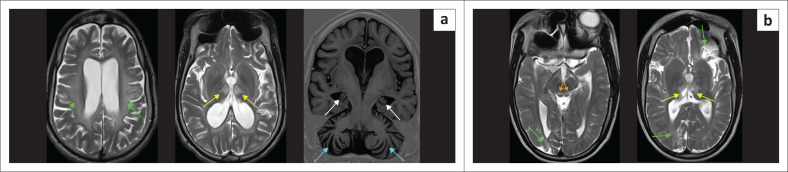
(a) (Left) Leigh syndrome because of complex IV disruption with *SURF-1* mutation. Confluent moderate T2-W hyperintensity in the white matter (green arrows), relatively sparing the cortex. The paramedian thalamic injury (yellow arrows) would be an atypical location for hypoxic-ischaemic brain injury. There is marked hippocampal atrophy (white arrows). Note the severe and disproportionate cerebellar atrophy with the appearance of bottom of fissure dysplasia pattern (cyan arrows). (b) (Right) Leigh syndrome. T2-W hyperintensity in the midbrain tectum (orange arrows) at the level of superior colliculi, paramedian thalami (yellow arrows) and multilobar spongiosis (green arrows). Again, the thalamic lesion location would be atypical of hypoxic-ischaemic brain injury, but the constellation of findings mimics the mixed-type hypoxic-ischaemic brain injury.

**FIGURE 11:**
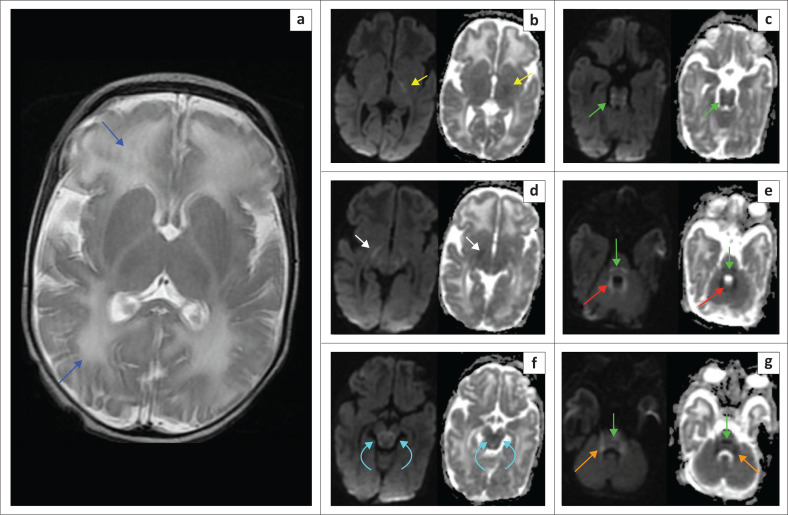
(a-g) A 5-day-old male with *Maple syrup urine disease*. Diffuse T2-W white matter hyperintensity (blue arrows) involving the juxta-ventricular and subcortical white matter confluently. Restricted diffusion is shown along the corticospinal tracts (yellow arrows), cerebral peduncles (white arrows), midbrain (cyan arrows), pontine tracts (green arrows), cerebellar peduncles (orange arrows) and around the fourth ventricle (red arrows). A reversible methyl peak may sometimes be seen at 0.9 ppm – 1.0 ppm on MRS.

**FIGURE 12:**
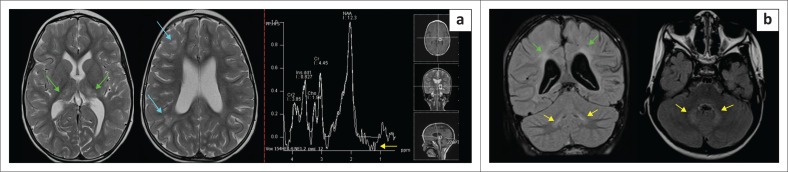
(a) (Left) *Molybdenum co-factor deficiency*. Atypical T2 hyperintensity in both atrophied thalami (green arrows), subcortical white matter (cyan arrows) and MRS shows a lactate peak (yellow arrow). There is an overall reduction in white matter volume. These patients can present with severe neonatal epileptic encephalopathy and directly mimic hypoxic-ischaemic encephalopathy. (b) (Right) *Sulfite oxidase deficiency* (SOD) in a 7-year-old child demonstrating extensive FLAIR hyperintensity of subcortical white matter (green arrows) sparing the overlying cortex. There is also hyperintense signal noted at the dentate nuclei of the cerebellum (yellow arrows). This pattern may simulate partial prolonged hypoxic-ischaemic brain injury with multiple watershed territory or border zone injuries. Cerebellar dentate nucleus injury may be seen in both SOD and hypoxic-ischaemic encephalopathy. Caudate nucleus injury is more common in SOD, and thalamic injury is less constant in SOD and vice versa in hypoxic-ischaemic encephalopathy.

**FIGURE 13:**
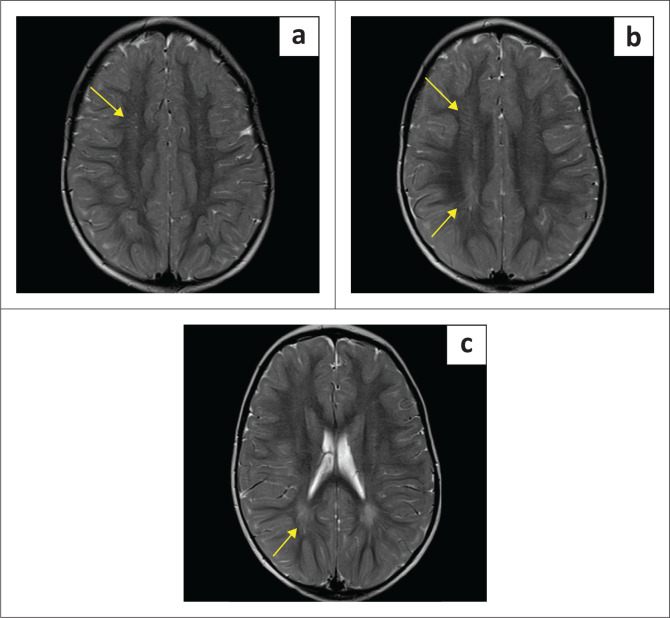
(a-c) A 21-month-old male child with a tigroid pattern (yellow arrows) of the centrum ovale white matter because of *Pelizeus Merzbacher-like disorder*.

**FIGURE 14:**
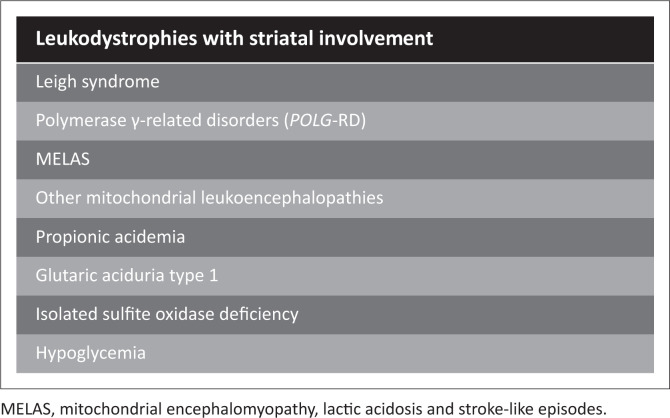
Leukodystrophies with striatal involvement that may mimic the Rolandic basal ganglia-thalamus pattern of hypoxic-ischaemic brain injury.

**FIGURE 15:**
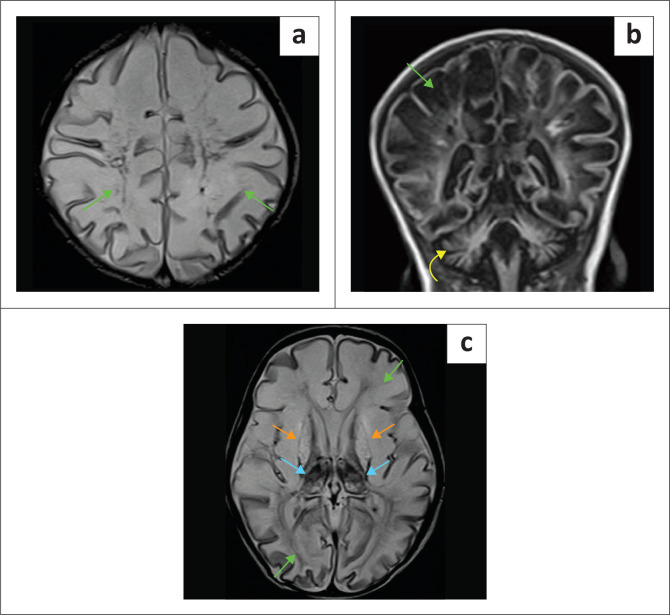
(a-c) *Isovaleric acidemia* in a neonate with diffuse white matter oedema (green arrows) and thalamic haemorrhagic degeneration (blue arrows). Note severe basal ganglia (orange arrows) and cerebellar (yellow arrow) injury. This simulates multicystic encephalomalacia.

**FIGURE 16:**
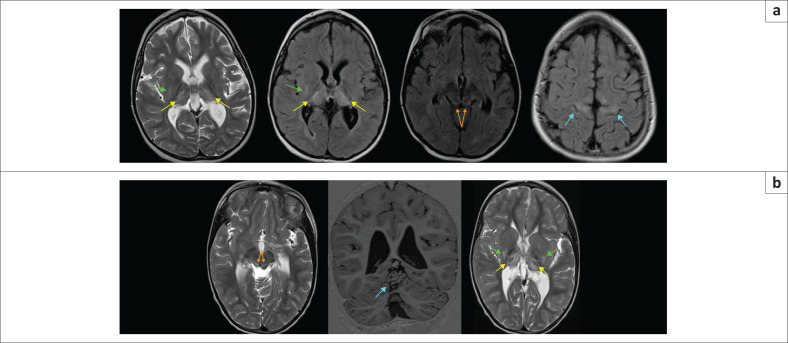
(a) (Top row) 5-year-old female patient with *polymerase* gamma-related disorders (*POLG-RD*) – T2-W and FLAIR hyperintensity in the perirolandic cortex (blue arrows), dorsal brainstem (orange arrows), thalami (yellow arrows) and putamen (green arrows). The initial presentation followed a preceding viral illness. The distribution of lesions mimics acute profound hypoxic-ischaemic brain injury with a basal ganglia-thalamus (BGT) pattern and perirolandic changes. When the differential diagnosis of hypoxic-ischaemic brain injury is suggested in the radiology report, medicolegal litigation is easily catapulted into action. (b) (Bottom row) Another patient with *POLG-RD* demonstrating T2-W hyperintensity in both thalami (yellow arrows), putamina (green arrows), midbrain tectum (orange arrows) and superior cerebellum (cyan arrow) are all features that mimic BGT or acute profound hypoxic-ischaemic brain injury.

**FIGURE 17:**
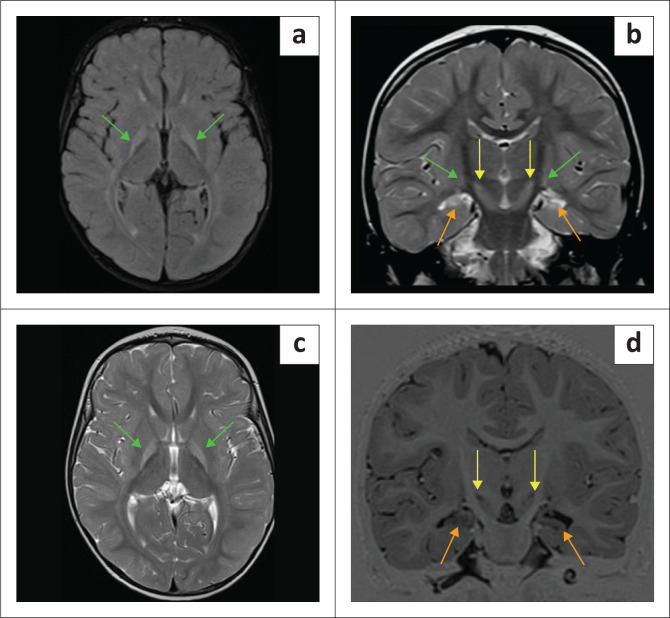
(a-d) Follow-up MRI in a 22-month-old male child with dyskinetic cerebral palsy and sensorineural hearing loss. There is severe bilateral hippocampal atrophy from intractable seizures. The hyperintense signal at the globi pallidi (green arrows), hippocampi (orange arrows) and both subthalamic nuclei (yellow arrows) are unchanged from the neonatal study and indicative of *kernicterus*. Note relative sparing of thalami and posterior putamina, which are expected to be involved in the Rolandic basal ganglia-thalamus subtype of hypoxic-ischaemic brain injury.

**FIGURE 18:**
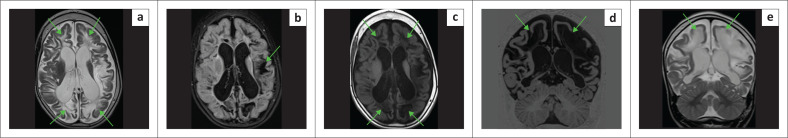
(a-e) A 5-year-old female with *L-2-hydroxyglutaric aciduria* demonstrating FLAIR hyperintensity of the cerebral white matter (green arrows). Cerebellar dentate nuclei are also commonly injured. The subcortical white matter is typically involved, sparing the periventricular white matter and simulating partial prolonged or parasagittal hypoxic-ischaemic brain injury. However, the relative sparing of the cortical ribbon is a feature against hypoxic-ischaemic brain injury. The reporting radiologist should look for this.

**FIGURE 19:**
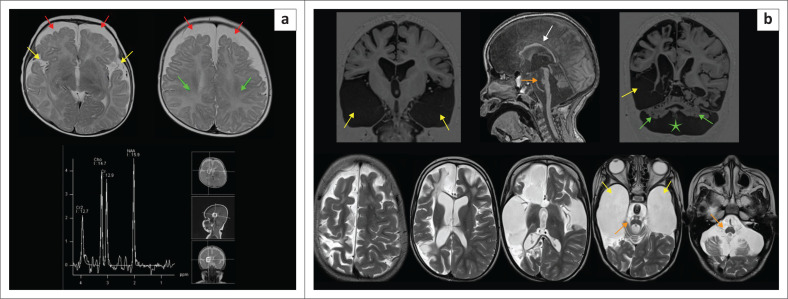
(a) (Left) *Glutaric aciduria type 1* in a 5-month-old male child. The MRS of the right frontal white matter and right lentiform nucleus revealed a decreased N-acetyl aspartate/creatine ratio, slightly increased choline/creatine ratio, and increased myoinositol/creatine ratio, compared with the age-matched control patients. Note diffuse T2-W white matter hyperintensity (green arrows), marked widening of the opercula (yellow arrows) and severe frontal lobe atrophy (red arrows). (b) (Right) A 5-year-old female with prior neonatal encephalopathy and multiple crises requiring admissions because of *Glutaric aciduria type 1*. Bilateral cystic encephalomalacia (yellow arrows), posterior fossa retrocerebellar cyst (green star) cerebellum (green arrows) injury, asymmetric cerebral encephalomalacia and severe thinning of the corpus callosum (white arrow) and brainstem (orange arrows).

**FIGURE 20:**
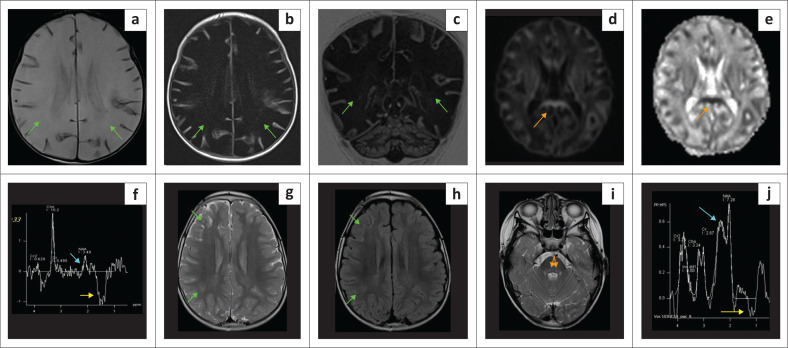
(a-f) A 15-day-old male with *ornithine transcarbamylase (OTC) deficiency*. The MRI reveals restricted diffusion in the corpus callosum (orange arrow), diffuse white matter interstitial oedema (green arrows), corresponding T1-W signal prolongation, with a glutamine-glutamate peak on the upstroke of the N-acetylaspartate peak (cyan arrow) and lactate peak (yellow arrow). Note the exaggerated choline: N-acetylaspartate ratio for neonates. (g-j) A 5-year-old male with a family history of a male sibling who passed away with a similar disorder. *Ornithine transcarbamylase deficiency* with subtle subcortical white matter changes (green arrows) with a glutamine-glutamate peak on the upstroke of the N-acetylaspartate peak (cyan arrow), lactate peak (yellow arrow) and central tegmental tract (orange arrows) hyperintensity.

Disorders of myelination result in abnormal white matter signals, which can mimic periventricular leukomalacia, which can also be a feature of HIBI in some neonates, although more commonly reported in premature children. These may include hypomyelinating disorders (Figure 13). Some leukodystrophies also have deep nuclear changes^16,17^ involving the corpus striatum, thalami as well as grey and white matter changes, as listed in Figure 14. When there is putaminal and thalamic involvement, it is difficult for the radiologist to differentiate these inborn errors of metabolism from the basal ganglia-thalamus pattern of HIBI. In the latter case, it is often associated with perirolandic cortex injury (the combination of which has previously been called the RBGT pattern^18^), especially when precipitated by a sentinel event or severe asphyxiation of the neonate. Such cases of HIBI are often associated with an acute profound pathophysiology where these high metabolic areas of the brain are subjected to a severe reduction in perfusion and oxygenation. The perirolandic sensorimotor cortex may also be abnormal in polymerase gamma-related disorders (*POLG-RD*) (Figure 16).^19^ The brainstem may also be injured along with these substrates (deep nuclear-brainstem pattern as per Volpe^20^). This is often life-threatening to both mother and child (e.g. uterine rupture or abruptio placentae). Therefore, in most cases, there is generally a correlative recording of such an event or stormy perinatal period in the patient’s notes. Furthermore, these cases of HIBI will have severe metabolic acidosis and the attendant cascade of inflammatory or other pathways activated, which is typically not seen in the first day of life in the case of most metabolic causes. Figures 15 to 20 highlight the typical imaging features of several metabolic disorders, which can be referred for diagnostic or medicolegal evaluation. These are examples of cases that we encountered with very similar imaging phenotypes to the broad subtypes of HIBI.

### Cerebrovascular causes

**FIGURE 21:**
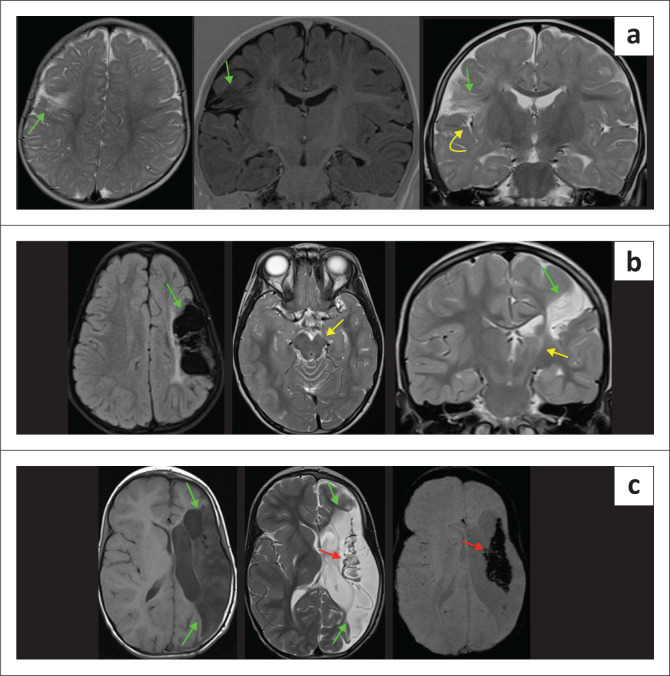
(a) A 5-month-old child with focal perinatal arterial ischaemic stroke due to subsegmental right middle cerebral artery (MCA) branch infarction (green arrows) and perisylvian spongiosis (yellow arrow). (b) A 6-year-old child diagnosed with perinatal arterial ischaemic stroke after a left MCA territory infarction (green arrows) and severe ipsilateral Wallerian degeneration (yellow arrows). (c) A 5-year-old child with haemiplegic cerebral palsy because of perinatal arterial ischaemic stroke following complete left MCA territory infarction (green arrows) with central haemorrhagic transformation (red arrows) and ex vacuo ventriculomegaly.

A few perinatal vascular causes must be considered as HIBI mimics. These may manifest as haemorrhagic or ischaemic phenomena. Perinatal arterial ischaemic stroke (PAIS) syndrome, which can present with NE, is defined as infarction in an arterial distribution between 28 weeks gestation and 28 days after birth.^20^ Focal clonic seizures are the typical initial presentation. Perinatal arterial ischaemic stroke may be difficult to differentiate clinically from HIE without acute and follow-up imaging, especially by MRI. The sustained injury is usually unilateral and limited to a single vascular territory, most commonly the middle cerebral artery territory. There can be marked variability (Figure 21) in the severity of PAIS, and there is also a propensity for haemorrhagic transformation.

**FIGURE 22:**
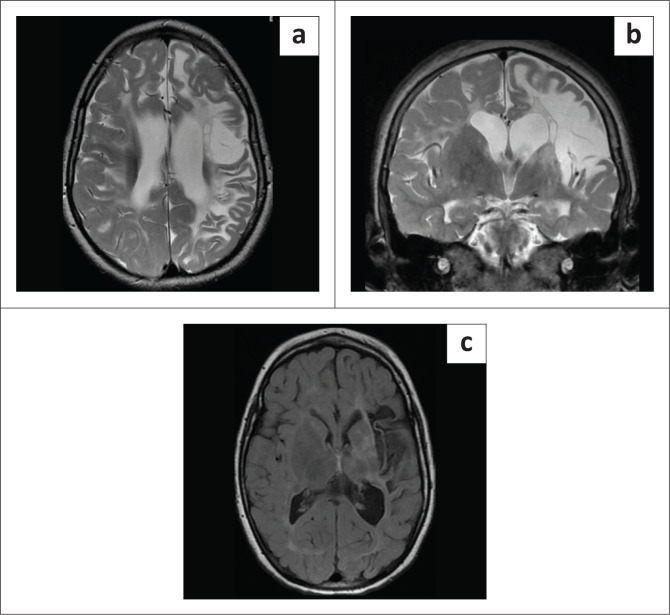
(a-c) Asymmetric encephalomalacia changes are noticed, particularly involving the left hemisphere, with relative sparing of the cortical ribbon. Spongiosis changes appear to involve the subcortical U fibres in the left hemisphere. There is a contiguous extension to involve the left insular cortex and the left lateral putamen. There is also T2 hyperintensity noticed in part of the caudate nucleus on each side. Secondary asymmetric porencephalic ventriculomegaly is present. This combination is unusual for hypoxic-ischaemic brain injury. On recommendation from the principal author, genetic testing was undertaken, which was positive for *Col4A1 mutation*.

The differential diagnosis for PAIS would include COL4A-1 and A-2 mutations (Figure 22). These children commonly may not have a history of perinatal encephalopathy, and there may be other clinical features such as cataracts.^21^ These collagen type IV genes encode for nearly all vascular basement membranes and have been shown to be associated with foetal intracranial haemorrhages, schizencephaly, porencephaly, and hydrocephalus.^22^

## Non-accidental injury

**FIGURE 23:**
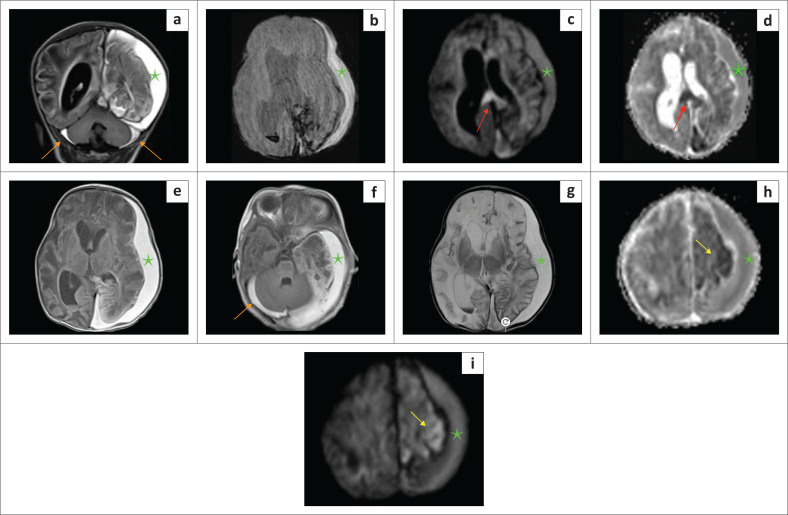
(a-i) A 9-day-old child with *suspected non-accidental injury*. Large left subdural haematoma (green star) noted. Note the mass effect with rightward midline displacement with smaller subdural haemorrhages (orange arrows) in the posterior fossa tracking around the cerebellum. There are ischaemic changes in the left hemisphere at the frontal lobe (yellow arrows) and the splenium of the corpus callosum (red arrows).

**FIGURE 24:**
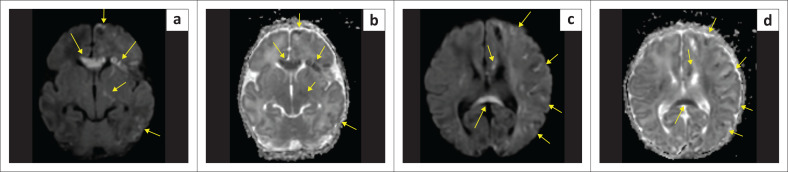
(a-d) A 10-day-old female child with suspected hypoxic-ischaemic brain injury. Restricted diffusion with ADC shortening is noted in the corpus callosum, cortex, internal capsule and deep nuclei (yellow arrows). The child did not demonstrate any acidosis, and there were violaceous lesions noted on the skin of the forearm. A diagnosis of *Incontinentia Pigmenti* was made.

One of the most difficult diagnoses a radiologist may be required to make is that of non-accidental injury (NAI), shown in Figure 23. In a neonate, the distinction of NAI from traumatic brain injury during delivery may sometimes be difficult. The combination of subdural haemorrhages, juxtacallosal shearing-type injuries and areas of ischemia are all highly suspicious for NAI. Furthermore, the location of subdural haemorrhages has been shown to be useful in distinguishing benign subdural haemorrhages (usually in the posterior fossa, around the tentorium cerebelli or around the occipital lobes) versus NAI (interhemispheric or over the cerebral convexities).^23^ Incontinentia pigmenti would be an important differential consideration, as shown in the companion case (Figure 24).

## Infections

**FIGURE 25:**
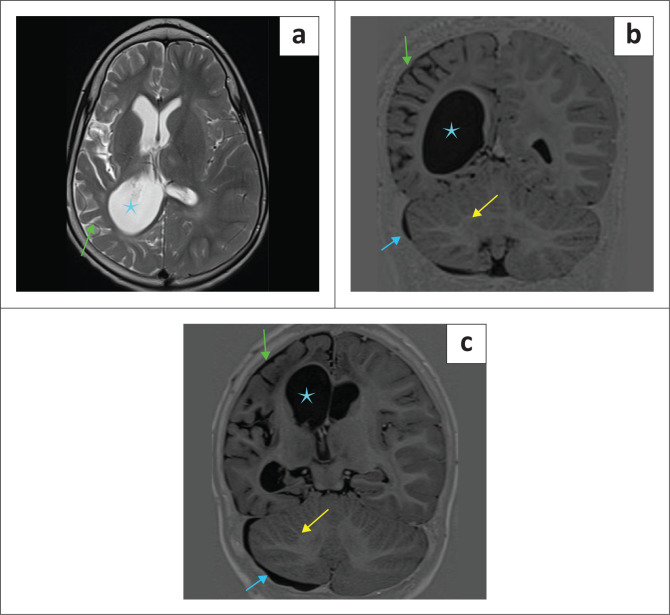
(a-c) *Intrauterine herpes simplex 2 infection* with unilateral cerebral (green arrows) and cerebellar (yellow arrows) atrophy, ventriculomegaly (cyan star) and subdural effusions (blue arrows).

**FIGURE 26:**
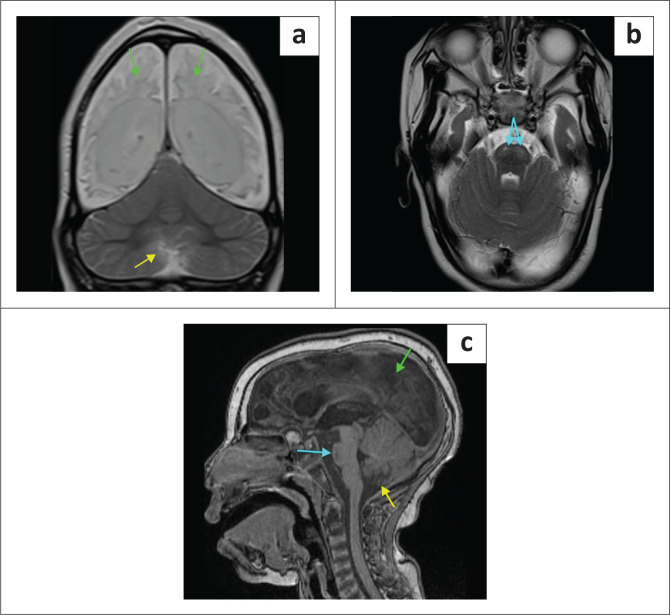
(a-c) A 3-year-old male child with a history of neonatal seizures. Clinical records revealed confirmed *Citrobacter cerebritis* with near total cerebral encephalomalacia (green arrows), ventral pontine (cyan arrows) and inferior cerebellar (yellow arrows) signal changes. The latter two findings are atypical of hypoxic-ischaemic brain injury.

**FIGURE 27:**
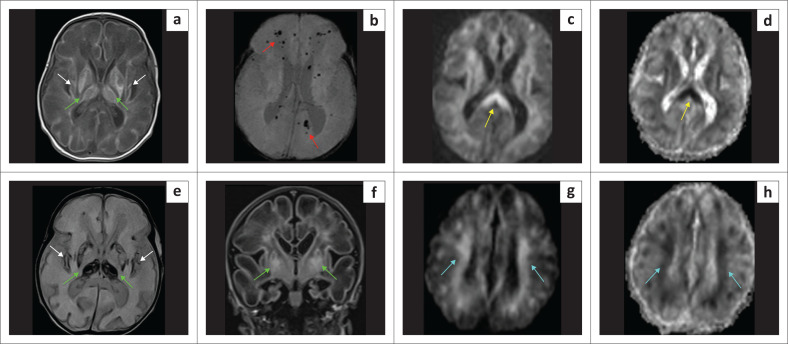
(a-h) Haemorrhagic infarction of the basal ganglia-thalami (green arrows), microhaemorrhages in the periventricular white matter (red arrows), insular cortex (white arrows) and restricted diffusion in the splenium (yellow arrows) and white matter (cyan arrows) due to complicated *Parechovirus leukoencephalitis*.

**FIGURE 28:**
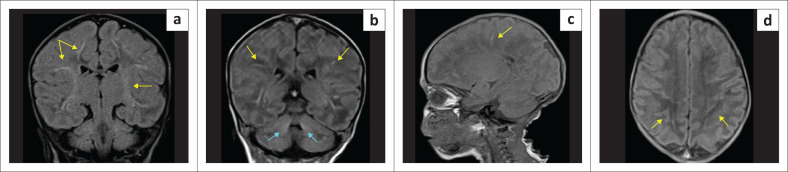
(a-d) Day-17-neonate with seizures because of neonatal *gram-negative meningo-encephalitis*. Note an unusual presentation with FLAIR and T1-W hyperintensity in the white matter (yellow arrows) extending along some perivascular spaces due to exudate. There is also mild signal change around the fourth ventricle and dentate nuclei (cyan arrows).

**FIGURE 29:**
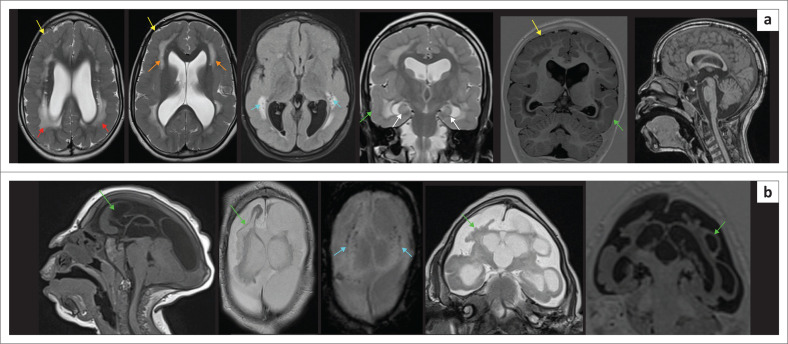
(a) Intrauterine cytomegalovirus *(CMV)* infection with a simplified gyral pattern (green arrows), polymicrogyria (yellow arrows), periventricular (red arrows) and subcortical (orange arrows) white matter hyperintensity with microcystic change (cyan arrows). Swollen hippocampi (white arrows) due to status epilepticus. Zellweger syndrome, Walker-Warburg syndrome and congenital lissencephaly type II are important differential considerations. (b) 7-month-old child with severe microcephaly. An MRI was performed to confirm suspected hypoxic-ischaemic brain injury for medicolegal evaluation. There is near complete destruction of the brain with periventricular calcification (cyan arrows). There is malformation of the cortex (green arrows) compatible with probable in utero *CMV* infection.

**FIGURE 30:**
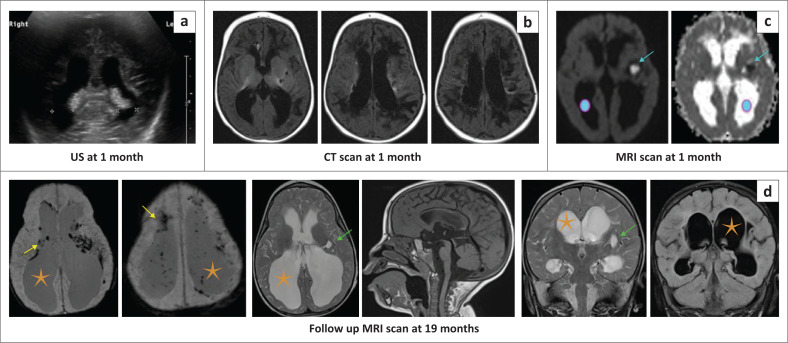
(a-d) In utero toxoplasmosis, rubella, cytomegalovirus, herpes (TORCH) (*cytomegalovirus*) infection with neonatal hydrocephalus and periventricular hyperechogenicity on sonar. Follow-up CT at 1 month shows periventricular calcification and increasing ventricular prominence. Putaminal restricted diffusion (cyan arrows) and slightly larger ventricles (O) were shown on the MRI studies performed at 1 month of age. Follow-up MRI at 19 months reveals more marked hydrocephalus (orange star), periventricular calcification (yellow arrows) and basal ganglia spongiosis (green arrow) related to prior secondary ischaemic injury. These sequential complications of *TORCH* infection are important in distinguishing it from hypoxic-ischaemic brain injury.

**FIGURE 31:**
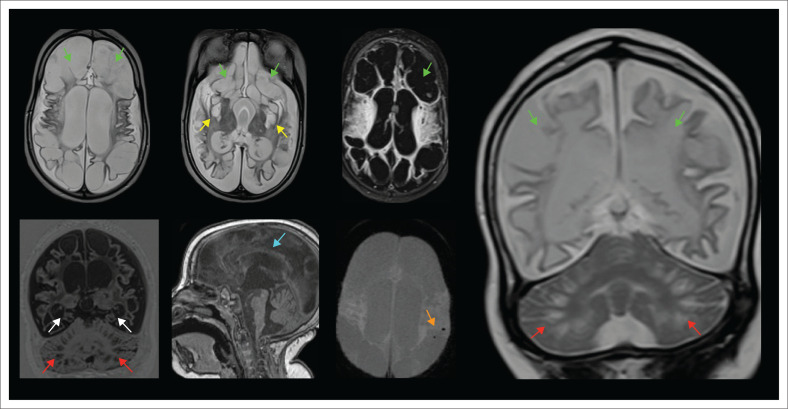
Destruction of the basal ganglia (yellow arrows), severe cystic encephalomalacia (green arrows), hippocampal atrophy (white arrows), corpus callosum thinning (cyan arrow) microhaemorrhages in the subcortical white matter (orange arrow) and cerebellar grey-white matter junction signal abnormalities simulating bottom of fissure dysplasia (red arrows) seen in a child with previous complicated *Parechovirus leukoencephalitis*.

**FIGURE 32:**
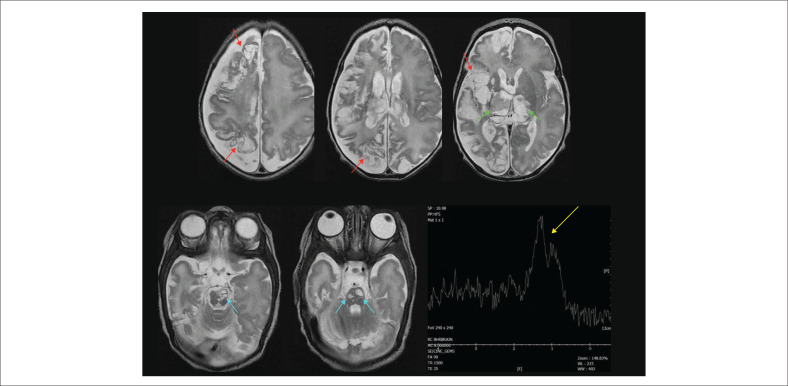
Twenty seven-day-old neonate with severe destruction of the right cerebral hemisphere (red arrows) because of *congenital infection* – exact aetiology unknown. The MRS shows reduced metabolite peaks except for a remarkably elevated broad peak from 0.8 ppm to 1.6 ppm because of molecular debris, including lactate (yellow arrow) from the severe brain injury. Note the vacuolation at the basal ganglia (green arrows) and brainstem (cyan arrows).

**FIGURE 33:**
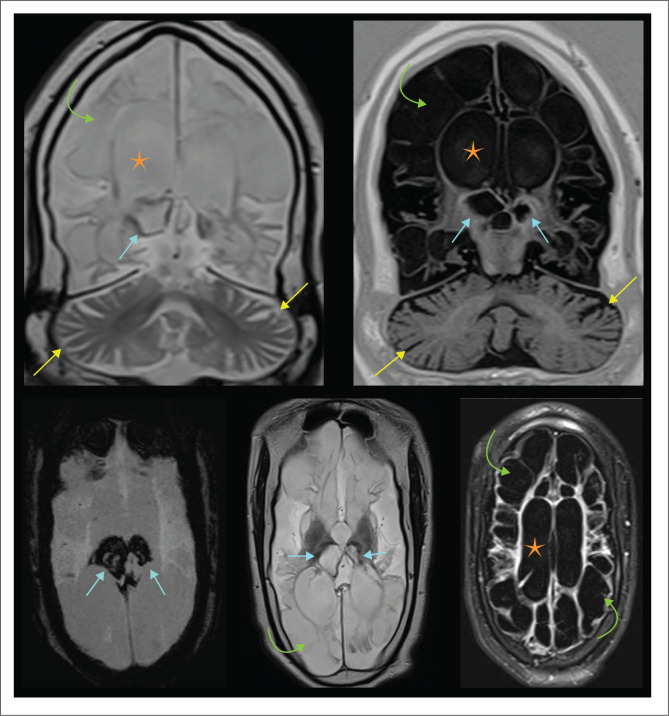
A 9-year-old male who suffered *in utero infection with severe multicystic encephalomalacia* (green arrows), ventriculomegaly (orange star) and cerebellar atrophy (yellow arrows). This was complicated by recurrent haemorrhagic acute necrotising encephalopathy. Note the haemorrhagic destruction of the thalami (cyan arrows).

Late in-utero and perinatal infection is an important cause of NE that may mimic HIE. TORCH infections are the predominant cause identified, as shown in Figures 25 to 33. Chorioamnionitis and subclinical maternal infection are possible causes of foetal and neonatal neurological injury.^24^ Placental evaluation is crucial for the identification of features of infection. Chronic villitis has been associated with basal ganglia-thalamus injuries and, combined with nucleated red blood cells and reduced placental maturation, may be associated with mixed basal ganglia-thalamus and watershed-type cerebral injuries.^24,25^ These undoubtedly mimic HIBI subtypes.

## Limitation

A major limitation lies in the accurate diagnosis of metabolic, chromosomal and genetic disorders because of the lack of available testing in resource-constrained settings. This is, however, improving as such laboratory and high-level gene sequencing facilities are becoming more accessible in South Africa.^26^ Some patients without clinical and laboratory results (no objective proof in the form of relevant serum or urine testing, genetics, etc.) have been excluded from the study. Including such cases, if clinical information was available, would add significantly to the findings of this study. Larger studies with available clinical data are therefore strongly encouraged.

## Conclusion

**FIGURE 34:**
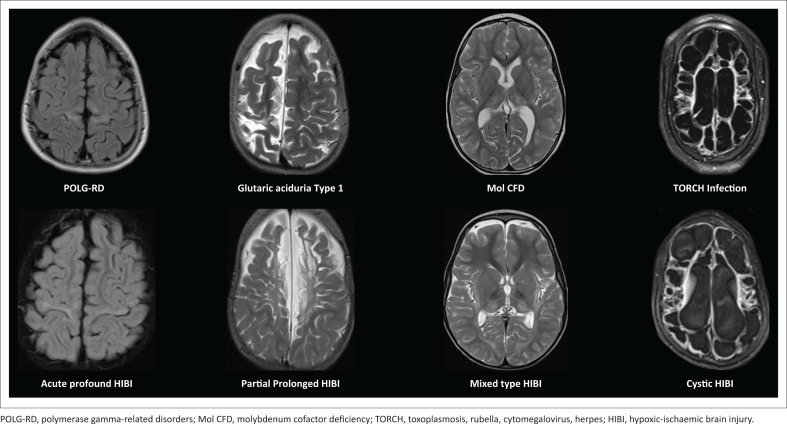
The diagnoses of the mystery cases are revealed with the bottom row listing the main subtypes of HIBI while the top row annotates mimicking conditions with often identical MRI patterns. The reporting radiologist will do well to keep this in mind when facing a challenging paediatric MRI study.

There are several potential radiological mimickers of neonatal HIE that can have a similar imaging appearance to HIBI (see Figure 34). It is prudent for the reporting radiologists to be aware of these alternate diagnoses, which may be associated with significant morbidity and have implications for medicolegal evaluations. To describe the clinical syndrome affecting these neonates, it has been proposed^27^ that a more descriptive terminology, such as NE, is preferable to a specific aetiology, such as HIE, which conveys a definitive known aetiology. By classifying the neonatal syndrome as NE, other causes (genetic conditions, infection, vascular, or multifactorial aetiology), will be considered and greater clarity is afforded to all parties involved. This, in effect, ensures that the parents are well-informed early on, and there is an opportunity for better patient outcomes with directed therapy where applicable.
